# Construction of a subunit-fusion nitrile hydratase and discovery of an innovative metal ion transfer pattern

**DOI:** 10.1038/srep19183

**Published:** 2016-01-12

**Authors:** Yuanyuan Xia, Wenjing Cui, Zhongmei Liu, Li Zhou, Youtian Cui, Michihiko Kobayashi, Zhemin Zhou

**Affiliations:** 1 Key Laboratory of Industrial Biotechnology, Ministry of Education, School of Biotechnology, Jiangnan University, Wuxi 214122, China; 2 Institute of Applied Biochemistry, and Graduate School of Life and Environmental Sciences, The University of Tsukuba, 1-1-1 Tennodai, Tsukuba, Ibaraki 305-8572, Japan

## Abstract

Metallochaperones are metal-binding proteins designed to deliver the appropriate metal to a target protein. The metal is usually transferred between different proteins. In this study, we discovered that metal was transferred between the same subunit of a mutant nitrile hydratase (NHase). Various “activator proteins” mediate the trafficking of metal ions into NHases. We constructed fusion NHases by fusing the β- and α-subunits and/or the “activator proteins” of the NHase from *Pseudomonas putida*. The fusion NHases exhibited higher thermostability and tolerance to high concentrations of the product amide. The mechanism of the cobalt incorporation changed from a self-subunit swapping pattern to an apoprotein-specific molecular chaperone pattern *in vivo* and a metallochaperone pattern *in vitro*. Notably, the cobalt transfer occurred between the same α-subunit in the metallochaperone pattern. These results not only demonstrated the superiority of fusion-type NHases, but also revealed an innovative metal ion transfer pattern in metalloprotein biosynthesis.

Metalloproteins have been intensively characterized for decades, and more than one third of all proteins are metalloproteins^1^. The roles mediated by metal ions in proteins include electron transfer, oxygen transport, gene regulation and structure stabilization^2^. Several general mechanisms of metallocenter biosynthesis were reported in a review^3^; one of them is metallochaperone delivery of a metal ion or metal-containing molecules. Metallochaperones are metal-binding proteins designed to deliver the appropriate metal ion or metal-containing molecules to a target protein. The target protein and the metallochaperone are usually different proteins, and the target protein exhibits ligand specificity and more affinity for the metal ion.

Nitrile hydratase (NHase, EC 4.2.1.84)^4^ is an enzyme catalysing the hydration of a broad scope of nitriles to the corresponding amides^5^. The enzyme is composed of α- and β-subunits and contains either a non-heme iron (Fe-NHase)^6^ or a non-corrin cobalt (Co-NHase)^7^,^8^. The trafficking of metal ions into NHases is mediated by the corresponding “activator proteins”^9^. The activators for Fe-NHases have been shown to act as metallochaperones^10^, whereas the “activator proteins” act as “self-subunit swapping chaperones” for cobalt incorporation into most Co-NHases^9^,^11^,^12^.

Self-subunit swapping is one of the post-translational maturation steps of the Co-NHase family^11^. The activator protein exists as a complex with the α-subunit of NHase, and cobalt incorporation into NHase is dependent on the α-subunit swapping between the cobalt-free α-subunit of NHase and the cobalt-containing α-subunit of the complex^9^. Self-subunit swapping is quite different from the currently known general mechanisms of metallocenter biosynthesis^9^,^11^,^13^, which were reported in a review by Kuchar and Hausinger^3^, as follows: (i) reversible metal-ion binding; (ii) metallochaperone delivery of a metal ion or cofactor; (iii) posttranslational modification creating a metal-binding site; (iv) synergistic binding of a metal with another component; (v) synthesis of metal-containing cofactors; (vi) metal incorporation coupled with electron transfer; and (vii) requirement for an apoprotein-specific molecular chaperone and so on^14^. Self-subunit swapping was first discovered in the L-NHase of *Rhodococcus rhodochrous* J1^9^, and subsequently, in the H-NHase of the same strain^11^, and it is speculated to occur in various other Co-NHases and an NHase family enzyme, thiocyanate hydrolase, which also contains a unique non-corrin cobalt centre with two post-translationally modified cysteine ligands^12^,^15^. “Self-subunit swapping chaperones” exhibit a surprising protein function in contrast with metallochaperones in metallocenter biosynthesis and molecular chaperones in protein folding^9^,^11^.

NHase is widely used in the industrial production of highly purified acrylamide and nicotinamide^4^. However, most NHases with high activity are unstable during industrial application^16^. For example, the NHases of *Pseudomonas chlororaphils* B23 and *Rhodococcus* sp. N-774 are stable under 20 °C^17^,^18^, and the NHase of *Rhodococcus rhodochrous* J1 is merely stable between 10 and 30 °C^19^. Because nitrile-hydration is an exothermic reaction, it is necessary to maintain low reaction temperature to stabilize the NHases by refrigeration, which usually causes enormous redundant energy cost^16^. Furthermore, the tolerance to high concentrations of the product amide is necessary in industrial manufacturing. Therefore, a more stable NHase with high activity and high tolerance is required for industrial manufacturing.

Subunit gene fusion can enhance protein stability^20^,^21^,^22^. Gene fusion is a strategy for making two or more previously distinct genes becoming fused into a single open reading frame. Furthermore, it has been reported that fusion events tend to optimize assembly by simplifying protein complex topologies^23^. Recently, a new gene structure of NHase in the eukaryote *Monosiga brevicollis* has been predicted^24^. The two usually separated NHase subunits (β- and α-subunits) are fused in one peptide in this NHase. The fusion NHase is predicted to have beneficial features, such as higher activity, higher stability or new substrate specificities^24^. Therefore, the gene fusion strategy could be an effective tool for improving the application of the NHase.

In this study, we constructed a new type of NHase with only one polypeptide by fusing the β- and α-subunits of the NHase from *Pseudomonas putida* NRRL-18668 (*P. putida*). The fusion NHase exhibited significantly higher thermostability and product tolerance than the wild type. The activator protein P14K was also necessary for the fusion NHase activation. In addition, P14K was further fused with the β- and α-subunits fusion NHase resulting in the formation of another fusion NHase. The cobalt incorporation mechanism of the NHase was changed due to these structure alterations. The complex α(P14K)_2_ (two P14Ks were combined with the α-subunit) played a role as metallochaperone to transfer a cobalt ion into the fusion NHases *in vitro*. The cobalt transfer occurred in the same α-subunit between cobalt-free NHase (apo-NHase) and the complex α(P14K)_2_, which revealed an innovative metal ion transfer pattern in metalloprotein biosynthesis. In addition, P14K most likely acts as an apoprotein-specific molecular chaperone for cobalt incorporation *in vivo*.

## Results

### Subunit gene fusion of the NHase

**Figure 1:**
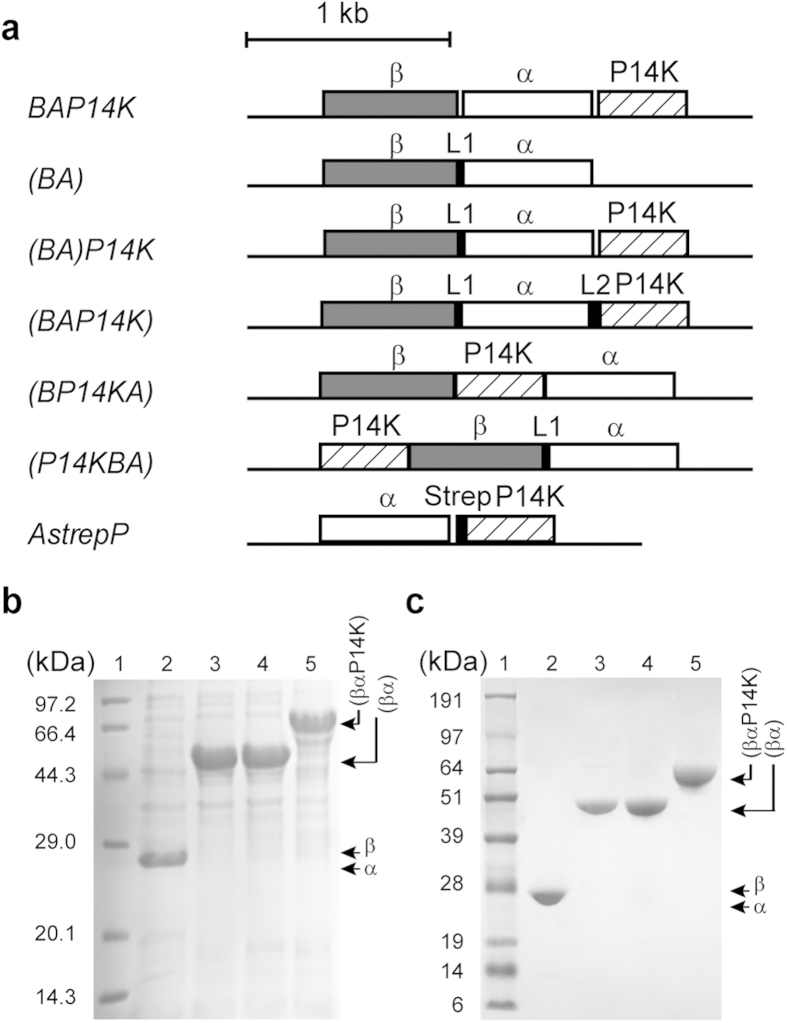
Construction, expression and purification of the recombinant NHases. (**a**) Genetic organization for the construction of a set of plasmids. (**b**) SDS-PAGE of a cell-free extract of each transformant. 1, marker; 2, wild-type NHase; 3, NHase-(BA); 4, NHase-(BA)P14K; 5, NHase-(BAP14K). (**c**) SDS-PAGE of the purified enzymes. 1, marker; 2, wild-type NHase; 3, NHase-(BA); 4, NHase-(BA)P14K; 5, NHase-(BAP14K).

The NHase from *P. putida* was previously constructed and over-expressed successfully in *Escherichia coli* BL21^25^. Based on the gene structure of *Monosiga brevicollis,* we constructed two mutant NHases with subunit gene fusion. The previously distinct β- and α- subunits genes (*B* and *A* genes) were fused with a linker (dipeptide, proline-glycine) into one gene *(BA)*; the fused gene *(BA)* was inserted into plasmid pET28a, resulting in pET28a*-(BA)* (Fig. 1a). Next, the activator gene *P14K* was inserted downstream from the mutant gene (*BA*), resulting in pET28a-*(BA)P14K*. The transformants harbouring pET28a-*(BA)* and pET28a-*(BA)P14K* were used to express NHase. As a result, NHase activity was detected, and each of the corresponding protein bands of the transfomants was obvious on the SDS-PAGE, indicating that the two fusion NHases were successfully expressed (Fig. 1b). Hereafter, the fusion NHases encoded by the gene *(BA)* and *(BA)P14K* are respectively referred to as NHase-(BA) and NHase-(BA)P14K.

### The characteristics of the fusion NHases

**Figure 2:**
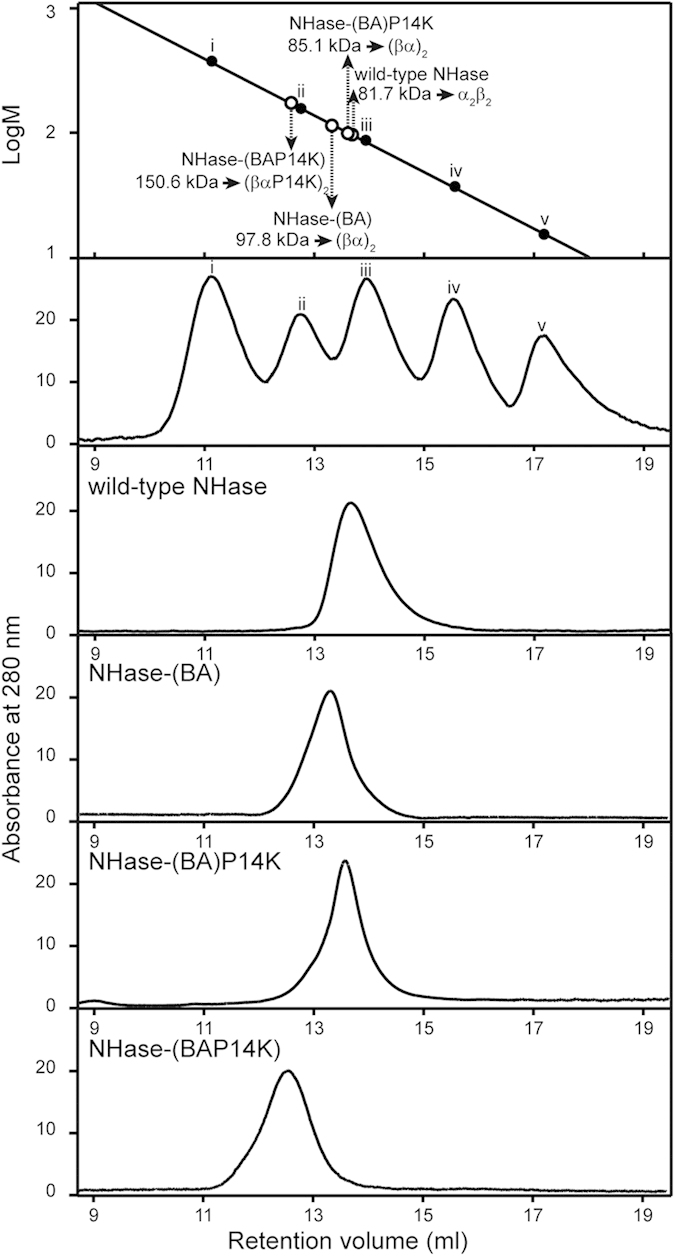
Determination of the molecular mass and structures of the fusion NHases. Marker proteins used for gel filtration: (i) glutamate dehydrogenase (yeast) (290 kDa); (ii) lactate dehydrogenase (pig heart) (142 kDa); (iii) enolase (yeast) (67 kDa); (iv) myokinase (yeast) (32 kDa); and (v) cytochrome c (horse heart) (12.4 kDa).

**Figure 3:**
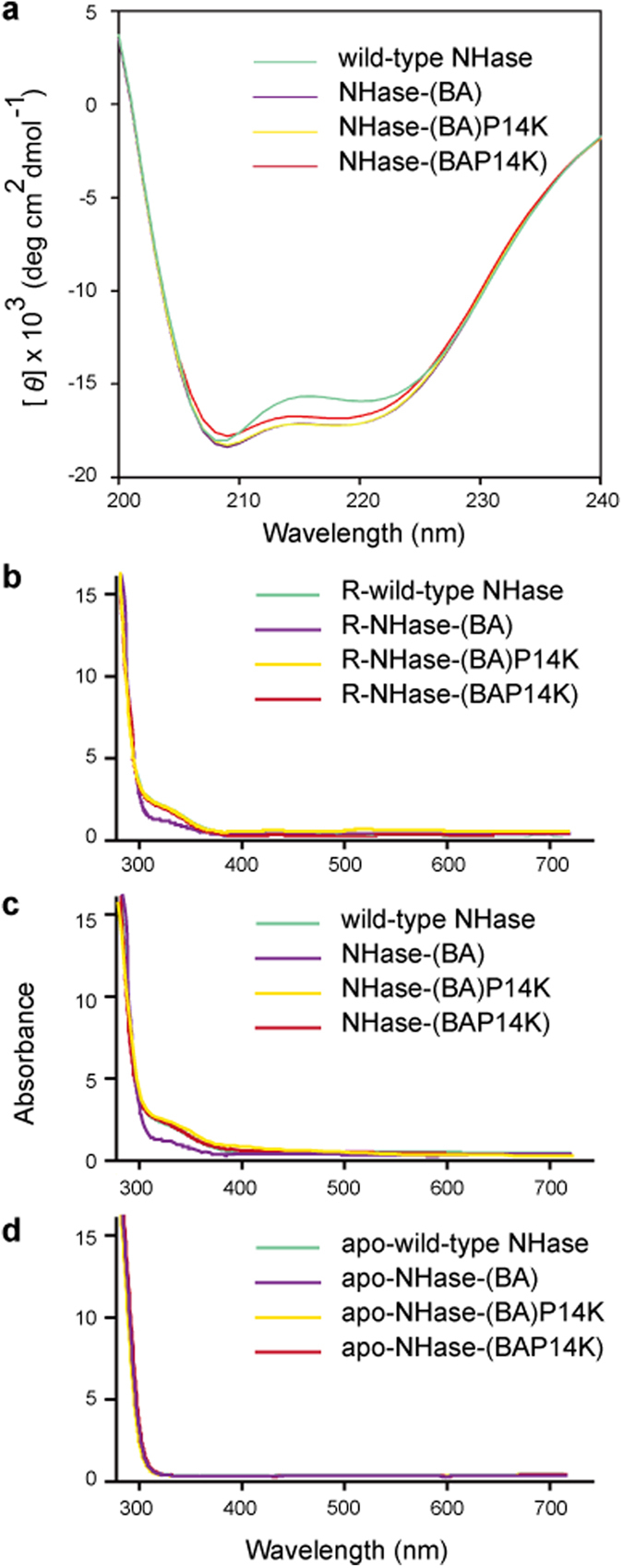
Far-UV CD (**a**) and UV-Vis absorption spectra (**b–d**) of the wild-type NHase and the fusion NHases.

NHase-(BA) and NHase-(BA)P14K were purified and characterized (Fig. 1c) and compared with the wild-type NHase. As shown in Table 1, the specific activity and *k*_cat_ of NHase-(BA)P14K were higher than those of wild-type NHase, demonstrating that the fusion of the β- and α-subunits increased the activity. The cobalt content of NHase-(BA) was approximately 14% of the NHase-(BA)P14K, indicating that P14K was also necessary for cobalt incorporation, even in the fusion NHase (Table 1). The Michaelis constant (*K*_m_ value) of both NHase-(BA) and NHase-(BA)P14K were higher (approximately 1.5-folds) than that of the wild-type NHase, indicating that the fusion of the β- and α-subunits might restrict the process of substrate binding to the active centre. MALDI-TOF mass analysis of the NHase-(BA)P14K indicated that the NHase-(BA)P14K is the full length of βα (Fig. S1). The wild-type NHase is a tetramer (α_2_β_2_); the molecular mass of the fusion enzymes was determined through gel-filtration chromatography. The molecular masses of NHase-(BA) and NHase-(BA)P14K were 97.8 kDa and 85.1 kDa, respectively (Fig. 2). Because the calculated molecular mass of fused βα is 49.2 kDa, both of the fusion NHases should be dimers [(βα)_2_]. Using far-UV circular dichroism (CD) spectra analysis, we conducted a detailed comparison of each property among the wild-type NHase, NHase-(BA) and NHase-(BA)P14K. The spectra of both NHase-(BA) and NHase-(BA)P14K were different from that of the wild-type NHase (Fig. 3a), indicating that the secondary structure of NHase-(BA) or NHase-(BA)P14K is not the same as that of the wild-type NHase.

### Construction of P14K to the fusion NHase

P14K was necessary for incorporation of cobalt in the wild-type NHase and the fusion NHases. We attempted to construct a three subunits fusion protein gene *(BAP14K)*, which further fused P14K to the (βα)-carboxyl terminus of NHase-(BA). The fused gene *(BAP14K)* was inserted into plasmid pET28a, resulting in pET28a-*(BAP14K)* (Fig. 1a). The transformant harbouring pET28a-*(BAP14K)* was used for NHase expression. As a result, the corresponding protein band of the mutant NHase was detected on the SDS-PAGE (Fig. 1b), indicating that the fusion NHase was successfully expressed. Hereafter, the fusion NHase encoded by the gene (*BAP14K*) is referred to as NHase-(BAP14K). The NHase-(BAP14K) was purified and characterized (Fig. 1c). As shown in Table 1, the specific activity and *k*_cat_ of NHase-(BAP14K) were higher than those of wild-type NHase, and the differences of the specific activity and *k*_cat_ between NHase-(BAP14K) and NHase-(BA)P14K were small. Compared with the wild-type NHase and NHase-(BA)P14K, the *K*_m_ value of NHase-(BAP14K) was the highest, indicating that the fusion of P14K further restricted substrate binding. The molecular mass of the NHase-(BAP14K) was determined through gel-filtration chromatography. The molecular mass of the NHase-(BAP14K) was 150.6 kDa. Because the calculated molecular mass of fusion βαP14K is 67.0 kDa, the NHase-(BAP14K) should be dimer [(βαP14K)_2_] (Fig. 2). The CD spectrum of the NHase-(BAP14K) was similar to that of the NHase-(BA)P14K (Fig. 3a), indicating that the secondary structure of the NHase-(BAP14K) is similar to that of the NHase-(BA)P14K but is different from that of the wild type.

### Effect of the P14K location on fusion NHase

Because the NHase-(BAP14K) exhibited higher NHase activity than the wild type, we studied the effect of the P14K location on the fusion NHase activity. We designed two more three subunits fusion NHase genes, *(BP14KA)* and *(P14KBA).* In *(BP14KA)*, the *P14K* gene was located between the β- and α-subunits genes; in *(P14KBA)*, the *P14K* gene was ahead of the (βα)-subunit gene (Fig. 1a). The transformants harbouring pET28a-*(BP14KA)* and pET28a-*(P14KBA)* were used to respectively express NHase-(BP14KA) and NHase-(P14KBA). The fusion NHases were purified and assayed. The activity of NHase-(BP14KA) was 148.6 U/mg, approximately 32% of that of the NHase-(BAP14K) (452.5 U/mg); the activity of NHase-(P14KBA) was 76.7 U/mg, approximately 17% of that of the NHase-(BAP14K). These findings demonstrated that the location of *P14K* significantly affected the fusion NHases activity. When the *P14K* located at the downstream of (βα)-carboxyl terminus, the fusion NHase showed the highest activity.

### Property comparison of the fusion NHases with the wild-type NHase

**Figure 4:**
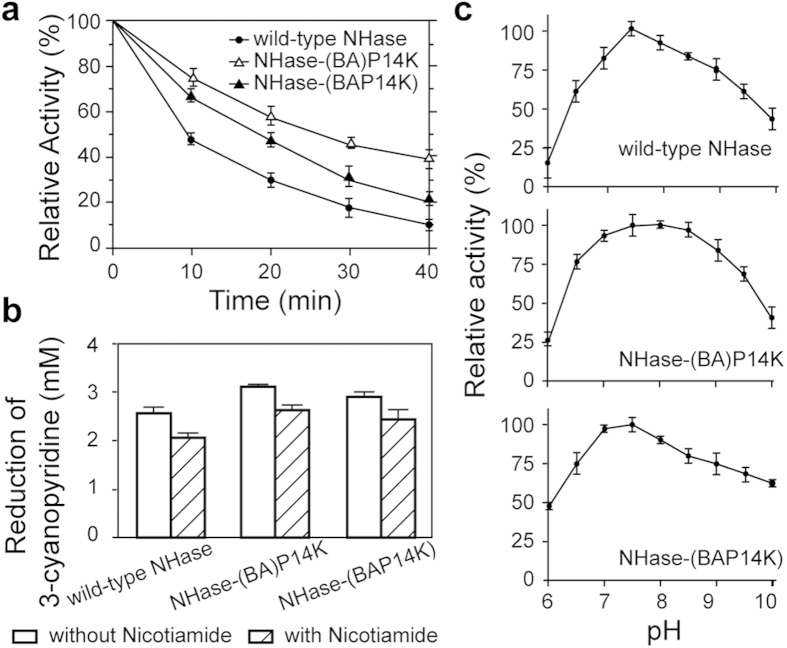
Property comparison of the fusion NHases with the wild-type NHase. (**a**) The analysis of the thermostability, (**b**) product tolerance and (**c**) pH optimum of the fusion NHases and the wild-type NHase.

The specific activity and *k*_cat_ of NHase-(BA)P14K and NHase-(BAP14K) were higher than those of the wild-type NHase; therefore, we further compared them in terms of their thermostability and product tolerance. The enzymes were incubated at 50°C in 10 mM potassium phosphate buffer (KPB) (pH 7.5, containing 0.5 mM dithiothreitol), and the activity of each NHase was measured every ten minutes. As shown in Fig. 4a, the half-life times of both NHase-(BA)P14K and NHase-(BAP14K) were respectively 2.8 and 2 times longer than that of the wild type, suggesting that NHase-(BA)P14K and NHase-(BAP14K) exhibit higher thermostability than that of the wild-type NHase.

In addition to thermostability, high accumulation of the product is beneficial for large-scale industrial production, which leads to energy savings and simplification of the downstream process^19^. To compare the tolerance of a high concentration amide of the wild-type and the fusion enzymes, we used nicotinamide to test the product tolerance of the NHases. The reaction was conducted in 20 mM 3-cyanopyridine (substrate) with and without 0.5 M nicotinamide (product) for 10 minutes. The reduction of 3-cyanopyridine in each reaction was measured (Fig. 4b), and the reduction ratio (the proportion of the reduced 3-cyanopyridine amount in the reaction with and without 0.5 M nicotinamide) was calculated. The reduction ratios of NHase-(BA)P14K and NHase-(BAP14K) were 0.86 and 0.83, respectively, which were higher than that of the wild type (0.80), indicating that the fusion NHases exhibited higher product tolerance than that of the wild type.

The physiologically optimal pH values of most NHases vary between 6.5 and 8.5, and NHase from *P. putida* has a broad activity maximum between pH 7.2 and 7.8^26^. The optimal pH of the fusion NHases were measured and compared with the wild type. As shown in Fig. 4c, the optimal pH of the wild type, NHase-(BA)P14K and NHase-(BAP14K) were 7.5, 8.0 and 7.5, respectively. Although NHase-(BAP14K) showed a similar stable range to that of the wild type, the stable range of NHase-(BA)P14K was broader, which was from 6.5 to 9.0, suggesting that this fusion might broaden the suitable pH scope of NHase.

### Activation of the fusion apo-NHase by α(P14K)_2_

P14K is necessary for cobalt incorporation into the NHase of *P. putida*^12^. P14K forms a complex α(P14K)_2_ with the α-subunit of the NHase. The incorporation of cobalt into the NHase is confirmed to be dependent on the α-subunit substitution between the cobalt-containing α(P14K)_2_ and apo-NHase, resulting in the formation of functional NHase^12^. P14K was also necessary for cobalt incorporation into the fusion NHases (Table 1). Here, we studied whether α(P14K)_2_ could activate the fusion NHases. The purified cobalt-containing α(P14K)_2_ was obtained by adding a Strep-tag ahead of P14K as described previously^12^ (Fig. 1a). The cobalt-free fusion NHases [apo-NHase-(BA), apo-NHase-(BA)P14K and apo-NHase-(BAP14K)] were purified from the culture in the absence of cobalt. The apo-wild-type NHase was also purified and was used as a control. The purified apo-wild-type NHase, apo-NHase-(BA), apo-NHase-(BA)P14K and apo-NHase-(BAP14K) were respectively mixed with the purified cobalt-containing α(P14K)_2_ and then incubated. The NHase activity in all of the mixtures increased as the incubation time increased, and the highest activity of the fusion NHases was observed in 1 hour. Each resultant NHase [R-wild-type NHase, R-NHase-(BA), R-NHase-(BA)P14K and R-NHase-(BAP14K)] was then purified from the mixtures and assayed. The NHase activity of the R-wild-type NHase was similar to that of the wild-type NHase, which accords with the principles of the self-subunit swapping reported previously^12^. Interestingly, the NHase activities of the R-NHase-(BA)P14K and R-NHase-(BAP14K) were 240.5 and 219.2 U/mg, respectively, which were approximately 50% of the corresponding cobalt-containing fusion NHases. The R-NHase-(BA) also exhibited 30% activity (147.2 U/mg) of the cobalt-containing fusion NHases (Table 1).

### Confirmation of cobalt transfer from α(P14K)_2_ to the fusion apo-NHases

Cobalt ion is necessary for NHase activity. The fusion apo-NHases were activated by α(P14K)_2_. These results indicate that cobalt ion was transferred from α(P14K)_2_ to the fusion apo-NHases. To confirm the cobalt transfer between α(P14K)_2_ and the fusion apo-NHases, UV-Vis absorption spectra analyses were performed. As shown in Fig. 3, all of the resultant enzymes exhibited an extra shoulder in the 300-to 350- nm region (Fig. 3b) similar to those of the NHases (cobalt-containing NHases) (Fig. 3c), whereas the extra shoulder was not detected in the apo-NHases (Fig. 3d). These findings suggested that R-NHase-(BA), R-NHase-(BA)P14K and R-NHase-(BAP14K) were cobalt containing enzymes. The cobalt content was determined, as shown in Table 1. R-NHase-(BA), R-NHase-(BA)P14K and R-NHase-(BAP14K) respectively contained 0.23 mol ion/mol (βα), 0.61 mol ion/mol (βα), and 0.62 mol ion/mol (βαP14K). Conversely, we also mixed apo-NHase, apo-NHase-(BA), apo-NHase-(BA)P14K and apo-NHase-(BAP14K) with cobalt ion and incubated. No significant activity was detected in these mixtures (data not shown), indicating that the fusion NHases could not be activated by mixing with cobalt ion *in vitro*, even for apo-NHase-(BAP14K), which contained the fragment of P14K.

### Investigation of Cysteine Modification in the fusion NHases

**Figure 5:**
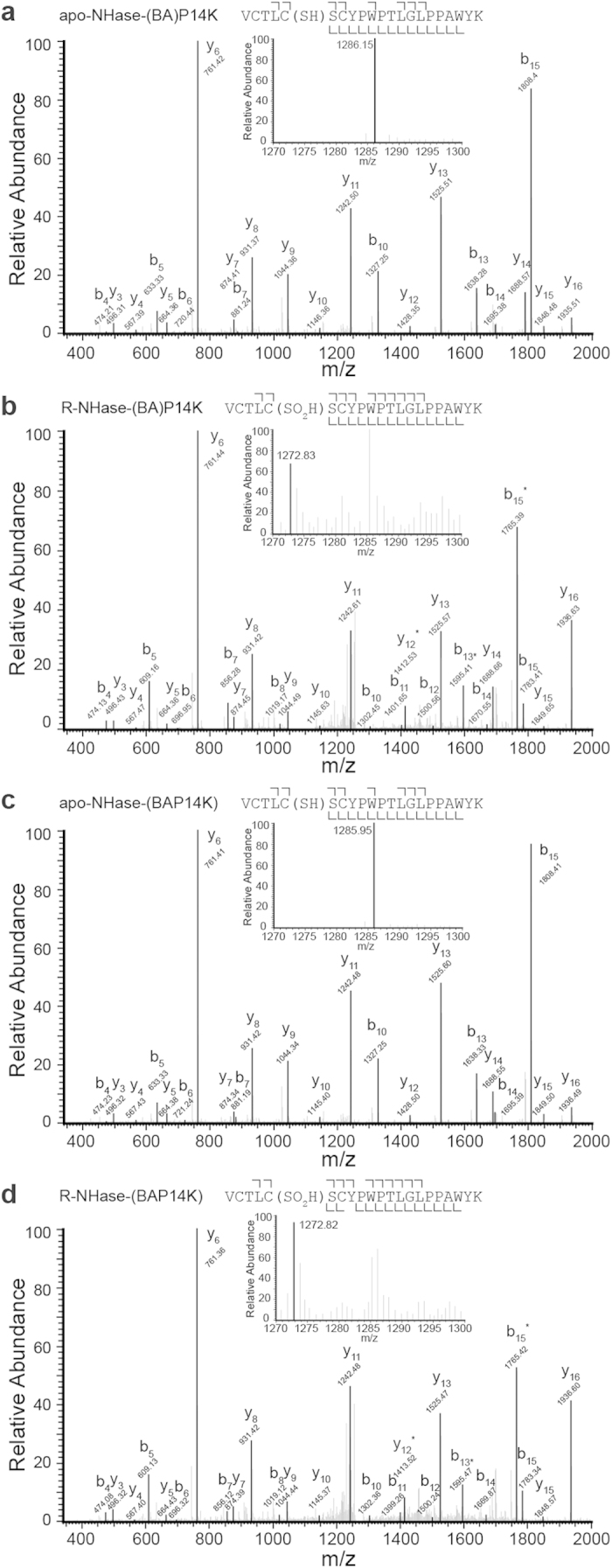
Identification of Cys355-SO_2_H by MS/MS analysis. Each doubly charged ion corresponding to the VK21 peptide of (**a**) apo-NHase-(BA)P14K, (**b**) R-NHase-(BA)P14K, (**c**) apo-NHase-(BAP14K), (**d**) R-NHase-(BAP14K) was analysed by a NanoLC-ESI-MS/MS system. The mass peaks with m/z 1286.15 and 1285.95 corresponding to the [M+2H]^2+^ ion of VK21 peptide with three CAM-cysteine (the calculated m/z value was 1285.94) were shown inside of (**a,c**); the mass peaks with m/z 1272.83, 1272.82 corresponds to the [M+2H]^2+^ ion of VK21 peptide with a Cys-SO2H and two CAM-cysteine (the calculated m/z value was 1273.44) were shown inside of **b,d**. The N- and C-terminal fragment ions at a peptide bond are respectively labelled in the figure as b and y. The b5 and b4 ions clearly indicated the Cys355 was oxidized to the sulfinic acid in R-NHase-(BA)P14K and R-NHase-(BAP14K), but not in apo-NHase-(BA)P14K and apo-NHase-(BAP14K). b* means b type ion after deamination and y* means y type ion after deamination.

Metal ion binding is responsible for the post-translational modification of the two cysteine in the α-subunit in NHase^3^,^26^. However, such modifications could not be observed in apo-NHase^26^. These results permitted us to speculate that the cysteine residues are modified in cobalt-containing fusion NHase, but not in apo-NHase. To clarify the cysteine oxidation state of active site before and after cobalt insertion, we analysed apo-NHase-(BA)P14K, apo-NHase-(BAP14K), R-NHase-(BA)P14K, and R-NHase-(BAP14K) by NanoLC-ESI-MS/MS. The enzymes were treated with trypsin after reduction and carboxamidomethylation. The molecular mass of the trypsin hydrolysed peptide containing all metal ligand residues V_351_CTLCSCYPWPTLGLPPAWYK_371_ (VK21) was measured. In the mass spectrum of the tryptic digest of apo-NHase-(BA)P14K and apo-NHase-(BAP14K) (Fig. 5a,c, inside), we found that the mass peak with m/z 1286.15 and 1285.95 respectively corresponded to the m/z value of the [M+2H]^2+^ ion of VK21 with three carboxamidomethyl (CAM-) cysteine (the calculated m/z value was 1285.94). However, in the mass spectrum of the tryptic digest of R-NHase-(BA)P14K and R-NHase-(BAP14K) (Fig. 5b,d, inside), we found that the most mass peak with m/z 1272.83 and 1272.82 respectively corresponded to the m/z value of the [M+2H]^2+^ ion of VK21 were both with a Cys-SO_2_H and two CAM-cysteine (the calculated m/z value was 1273.44). To identify the modified residue(s), we performed MS/MS sequencing of the ions with the m/z 1286.15, 1272.83, 1285.95 and 1272.82. The spectra were in good agreement with the predicted fragmentation pattern of VK21 with Cys355-SO_2_H in R-NHase-(BA)P14K and R-NHase-(BAP14K), and CAM-Cys355 in apo-NHase-(BA)P14K and apo-NHase-(BAP14K). Although the occurrence of Cys-SOH modification has not been confirmed because of its chemical instability^27^, these results strongly suggest that the oxidized cysteine residues exist in the two fusion NHases after cobalt insertion.

## Discussion

**Figure 6:**
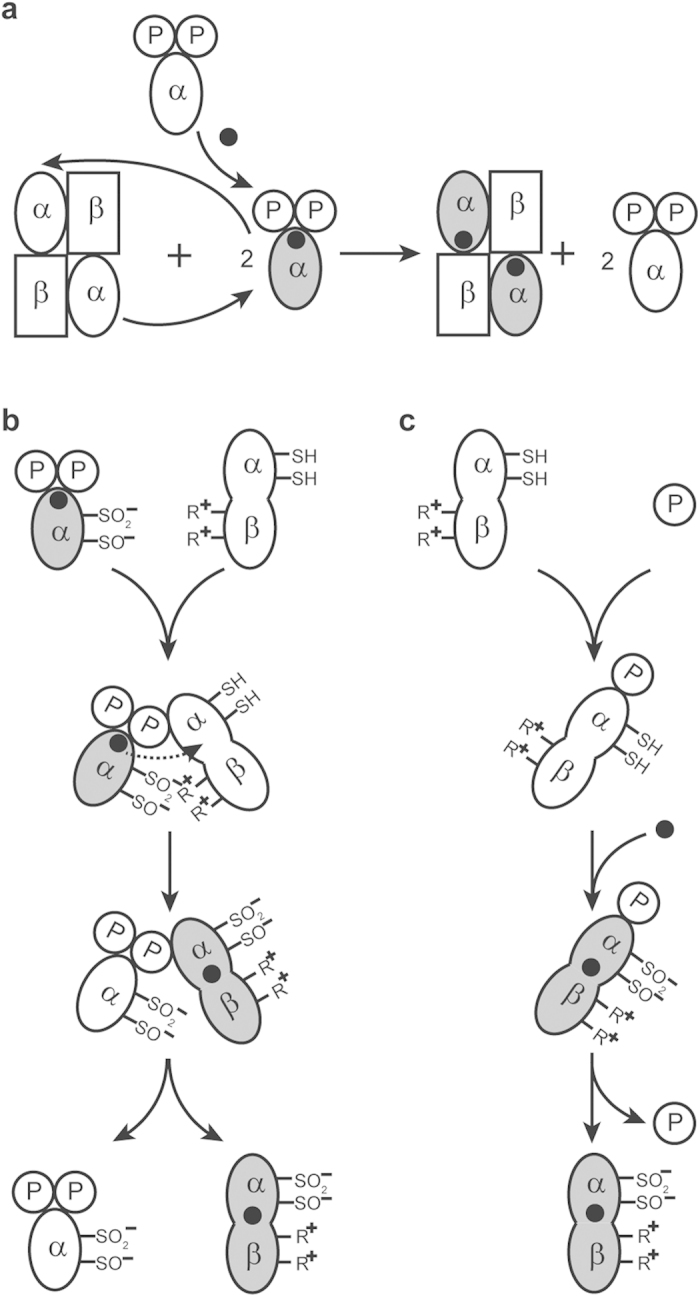
Proposed mechanism of cobalt incorporation into the fusion NHase. (**a**) Self-subunit swapping for cobalt incorporation into the wild-type NHase. (**b**) Cobalt transfer from α(P14K)_2_ to apo-NHase-(BA)P14K *in vitro*. The apo α-subunit in apo-NHase-(BA)P14K approaches and binds to the recognition (binding) site of P14K during self-subunit swapping^11^, and then the β-subunit (of apo-NHase-(BA)P14K) and the cobalt-containing α-subunit (of α(P14K)_2_) are attracted through the electrostatic interaction and associate with each other to form an intermediate complex. Instead of α-subunit exchange between the two proteins, cobalt ion direct transfer occurs. Oxidation of the two cysteine is responsible for cobalt binding, resulting in active NHase-(BA)P14K. (**c**) Cobalt incorporation into the fusion apo-NHase-(BA)P14K *in vivo*. P14K directly contacts with the α-subunit domain of the fusion βα protein because the binding sites in the α-subunit are free due to the fusion, resulting in an intermediate complex for cobalt insertion and the two cysteine oxidation. In these models, one fusion βα protein is used to show apo-NHase-(BA)P14K, the change of the location of the two arginine is used to demonstrate further folding after cobalt incorporation.

It has been confirmed that cobalt incorporation into the NHase of *P. putida* is dependent on the α-subunit swapping between the cobalt-free α-subunit of the apo-NHase and the cobalt-containing α-subunit of the α(P14K)_2_^12^ (Fig. 6a). For the fusion NHases, P14K was also necessary for the fusion NHases activation, and cobalt ion was found to be transferred from α(P14K)_2_ into the fusion NHases *in vitro* (Table 1). It is unlikely that α-subunit swapping occurs between the fusion NHases and α(P14K)_2_ because the α-subunit is fused with the β-subunit as one polypeptide in the fusion NHase. Therefore, it is likely that the cobalt ion in the α-subunit of α(P14K)_2_ was directly transferred into the α-subunit domain of the fusion NHases. In this process, α(P14K)_2_ probably acted as a metallochaperone for the cobalt ion transfer. The cobalt ion was transferred between the same two α-subunits.

Metal ions in both Fe-NHase and Co-NHase are located in their α-subunits, which share a characteristic metal binding motif (CXLC(SO_2_H)SC(SOH)) containing two oxidized cysteine residues: cysteine-sulfinic acid (Cys-SO_2_H) and cysteine-sulfenic acid (Cys-SOH)^4^,^28^,^29^,^30^. The oxidized cysteine residues are essential for NHase activity^9^,^31^, indicating that the two cysteine residues in the fusion NHases should be oxidized. The corresponding two oxidized cysteine residues are found to exist in the α-subunit of αe_2_ (the complex used for the self-subunit swapping of L-NHases in *R. rhodochrous* J1)^9^, suggesting that the two cysteine residues in α(P14K)_2_ should be oxidized. Metallochaperones are metal-binding proteins designed to deliver the appropriate metal ion or metal cofactor to their highly specific targets^3^. The metal ion or metal cofactor is usually transferred between two different proteins, and the transfer is dependent on the ligand’s specificity and affinity for the metal ion or metal cofactor, such as copper metallochaperone transferring copper ions to ATPase and superoxide dismutase^32^,^33^,^34^,^35^, and nickel metallochaperones transferring nickel ions to urease and hydrogenase^36^,^37^,^38^. During these processes, the copper metallochaperones are completely different to ATPase and superoxide dismutase, and the nickel metallochaperones are completely different to urease and hydrogenase, the target proteins should exhibit higher ligand’s specificity and affinity than metallochaperones. However, though α(P14K)_2_ and NHase are two different proteins, the α subunits in α(P14K)_2_ and in the fusion NHase have the same amino acid sequence and should have the same cobalt ion ligands. Therefore, we discovered a metal ion transfer pattern for metalloprotein biosynthesis, in which a metal ion is transported between the same protein subunits in different protein complexes.

It is mysterious that the cobalt ion transfers within the same protein with the same ligand environment. This phenomenon may be related to the structure of the active site of NHase. The post-translationally oxidized Cys-SO_2_H and Cys-SOH have deprotonated Cys-SO_2_^−^ and Cys-SO^−^ structures, respectively, and the deprotonated Cys-SO_2_^−^ and Cys-SO^−^ in the α-subunit form salt bridges with two arginine of the β-subunit of the NHase (Fig. S2)^9^. Only the electrostatic force is needed for the salt triggering self-subunit swapping^9^. The corresponding salt bridges should not exist in α(P14K)_2_ because P14K is shorter than the β-subunit, lacking the corresponding arginine (Fig. S3). The salt bridges probably influenced the ligand environment of the cobalt ion, resulting in a higher affinity for the cobalt ion. Therefore, when α(P14K)_2_ is mixed with the fusion NHases, the cobalt ion is transferred from the α-subunit of α(P14K)_2_ to the α-subunit domain of the fusion NHases. The cobalt transfer process is associated with cysteine oxidation due to the cysteine oxidation function of the self-subunit swapping chaperone^9^. Such a process is shown in Fig. 6b.

Whereas the apo-NHase-(BA)P14K was activated by α(P14K)_2_ up to 50% of the fusion NHases, apo-NHase-(BA) was activated only up to 30% activity (Table 1). These findings indicate that the structure of apo-NHase-(BA)P14K might be different from that of the apo-NHase-(BA), and apo-NHase-(BA)P14K is more suitable for cobalt incorporation by α(P14K)_2_. The difference between apo-NHase-(BA) and apo-NHase-(BA)P14K is in the presence or absence of the *P14K* gene, indicating that protein P14K effects the fusion NHase structure, and we can speculate that P14K also likely acts as a molecular chaperone for NHase folding.

Although the cobalt ion was transferred from α(P14K)_2_ into the fusion NHases *in vitro* (Table 1), this process should not exist *in vivo* because α(P14K)_2_ can not be formed in a cell because the β- and α-subunits are fused together into one protein. Because P14K is necessary for activating the expression of fusion NHase and acts as a molecular chaperone for NHase folding, we proposed a post-translational process for NHase-(BA)P14K *in vivo*. As shown in Fig. 6c, after translation of the *BA* and *P14K* genes, P14K contacts with the α-subunit domain of the fusion βα protein, and a cobalt ion is inserted into the α-subunit domain with the assistance of P14K. Protein folding occurs with the assistance of P14K after cobalt incorporation, resulting in the formation of functional fusion NHase. P14K dissociates from the fusion NHase during the post-translational process^25^. In this process, P14K probably acts as an apoprotein-specific molecular chaperone^3^ for cobalt incorporation into the fusion NHases, such as GroEL, Hsp70, and Hsp40, which bind to and prevent misfolding or stimulate refolding of a wide range of proteins^39^.

Because P14K is first contacted with the α-subunit domain of the fused βα protein for cobalt incorporation into the fusion NHases, a question rises of why this process could not occur in the wild-type NHase. P14K and other self-subunit swapping chaperones exhibit significant sequence similarity to the corresponding NHase β-subunit (approximately 30% homology) (Fig. S3), and both of them connect with the α-subunit. The β-subunit and P14K might have the same binding sites in the α-subunit. A competition for α-subunit binding occurs during the post-translational process of the wild-type NHase. As a result, both apo-NHase and α(P14K)_2_ are formed, followed by self-subunit swapping for NHase biosynthesis. In this case, P14K could not bind to the βα, because the α-subunit binding sites are occupied by the β-subunit. For the NHase-(BA)P14K, the β-subunit is fused with the α-subunit and the α-subunit binding sites become free. Therefore, P14K directly binds to the α-subunit binding sites of the fusion βα for cobalt incorporation.

We have discovered that the cobalt incorporation into most of the Co-NHases is dependent on self-subunit swapping both *in vitro* and *in vivo*^9^,^11^, whereas the cobalt ion in the α-subunit of α(P14K)_2_ is directly transferred into the α-subunit domain of the fusion NHases instead of self-subunit swapping *in vitro* (Table 1). Therefore, another question rises of why the direct transfer could not occur during the biosynthesis of the wild-type NHase. It is possible that both of the mechanisms occur in the wild-type NHase biosynthesis process; the prior mechanism would be self-subunit swapping. The binding of cobalt with the α-subunit is tighter than the binding of self-subunit swapping chaperone with the α-subunit, so α-subunit exchange is easier than cobalt direct transfer. Therefore, although both mechanisms occur in the wild-type NHase biosynthesis process, self-subunit swapping might be prior to cobalt direct transfer. The stand-by mechanism of the cobalt directly transfer may have a partial function for cobalt incorporation. As a result, apo-NHase-(BA)P14K and apo-NHase-(BAP14K) are only partially activated by α(P14K)_2_ (Table 1). In addition, the self-subunit swapping chaperone plays an important role in cobalt ion incorporation into the α-subunit. It has been reported that the flexibility of the C terminal domain of P14K is one of the key factors for cobalt incorporation into the α-subunit^40^; the fusion NHases encoded by genes *BP14KA* and *P14KBA* would affect the flexibility of the C terminal domain of P14K because there is an α- or β-subunit located in the C terminal domain of P14K, which reduces the flexibility. This may be one reason that the two fusion NHases exhibited lower activity.

Gene fusion substantially forces a pair of proteins to permanently interact with each other, and the fusion event tends to optimize the assembly by simplifying protein complex topologies^23^. Here through a gene fusion strategy, we constructed two fusion NHases with superior properties. The mechanism for cobalt incorporation was altered along with this molecular evolution *in vivo*, and we discovered a metal ion transfer pattern that occurs between the same proteins *in vitro*, which revealed an unexpected behaviour of a metallochaperone. The mechanism of cobalt incorporation into NHase is fantastic and variable, and the details should be further investigated.

## Methods

### Bacterial strains and plasmids

The *P. putida* wild-type NHase encoding gene *ABP14K* was obtained from *P. putida*^12^. Plasmids pET-24a (+) and pET-28a (+) were used as vectors and *E*. coli BL21 (DE3) was used for over-expression.

### Construction of plasmids

Oligonucleotide primers used in this study were shown in Table S1. The plasmid pET24a-*BAP14K* was used as the template, and B-*Nde* I-up and P-*Hin*d III-down primers were designed for introduction of *Nde* I and *Hin*d III restriction sites, respectively. The amplified DNA fragment was purified using a PCR purification kit (Promega) and digested with *Nde* I and *Hin*d III, ligated into the *Nde* I and *Hin*d III sites of pET-28a (+), then transformed into *E.coli* JM109 and sequenced. If the sequence was correct, the plasmid extract from *E.coli* JM109 was transformed into *E.coli* BL21 (DE3). The recombinant plasmids (pET28a-*(BA)P14K* and pET28a-*(BAP14K)*) were constructed by an overlap extension PCR protocol based on pET28a-*BAP14K*. To construct pET28a-*(BA)P14K* and pET28a-*(BAP14K)*, primer pairs Linker1-up and Linker1-down were used to connect the β- and α-subunits, and Linker2-up and Linker2-down were used to connect α- and P14K-subunits. Next, pET28a-*(BA)* was constructed with the primer pairs B-*Nde* I-up and A-*Hin*d III-down amplifying *(BA)* from pET28a-*(BA)P14K*, and the PCR product and pET-28a (+) were both digested with *Nde* I and *Hin*d III after 4 hours. The purified DNA fragment was then ligated into the *Nde* I and *Hin*d III sites of pET-28a (+). The recombinant plasmids (pET28a-*(BP14KA)* and pET28a-*(P14KBA)*) were respectively constructed by an overlap extension PCR protocol based on pET28a-*(BA)* with the PCR product P14K-1 and P14K-2 as primers. The PCR product P14K-1 was amplified with the primer pairs B(P)A-up and B(P)A-down, and P14K-2 was amplified with the primer pairs (P)BA-up and (P)BA-down with the pET24a-*BAP14K* as template.

### Media and culture conditions

The recombinant *E.coli* for expression of the enzyme was firstly cultivated in 10 ml of liquid 2YT medium containing 50 μg/ml kanamycin at 37°C, then cultivated in 500 ml of liquid 2YT medium in a 2 litre flask containing 50 μg/ml kanamycin at 37°C with shaking at 200 rpm. When the optical density at 600 nm (OD_600_) of the culture reached 0.8, isopropyl β-D-1-thiogalactoside (IPTG) was added to a final concentration of 0.4 mM to induce NHase expression, and CoCl_2_.6H_2_O was added to a final concentration of 0.05 g/l to obtain mature NHase. The culture was subsequently incubated at 24°C for 16 h.

### Purification and molecular mass determination of NHase proteins

All purification steps were performed at 0–4 °C. Cells were harvested by centrifugation at 6,000 × *g* for 15 min, resuspended in 10 mM KPB (containing 0.5 mM dithiothreitol, pH 7.4), and lysed by sonication on ice. The supernatants were removed by centrifugation at 15,000×*g* for 15 min and filtered through a 0.22-μm pore-size filter. For purification of proteins without any tag, a DEAE-Sephacel column (3 × 5 ml) (GE Healthcare UK Ltd.) was used. First, the column was equilibrated with 10 mM KPB, and the protein was eluted with a linear gradient from 0 to 0.5 M KCl in KPB. The active fractions were collected, concentrated to 1 ml by ultrafiltration and applied to a Superdex^TM^ 200 10/300 GL column (GE Healthcare UK Ltd.), and equilibrated with 10 mM KPB, with a flow rate of 0.5 ml/min. For purification of His-tagged enzymes, the filtrate was applied to a HisTrap HP chromatography column (GE Healthcare) using an AKTA purifier (GE Healthcare, Houston, TX). The column was equilibrated with buffer A (50 mM phosphate buffer, 0.3 M NaCl and 20 mM imidazole, pH 7.4), and the bound proteins were eluted with buffer B (50 mM phosphate buffer, 0.3 M NaCl and 500 mM imidazole, pH 7.4). Likewise, for purification of Strep-tagged enzymes, a StrepTrap HP column (GE Healthcare UK Ltd.) was equilibrated with binding buffer (20 mM Na_2_HPO_4_·12H_2_O, 280 mM NaCl, 6 mM KCl, pH 7.4). After injecting supernatant onto the column and incubating for half an hour, we eluted proteins with elution buffer (binding buffer containing 25 mM desthiobiotin, pH 7.4). The active fractions were collected, and the next step of gel filtration was the same as mentioned above. The purified proteins were analysed by sodium dodecyl sulphate-polyacrylamide gel electrophoresis (SDS-PAGE) and the protein concentration was quantified by the Bradford method^41^.

To determine the molecular masses and structures of NHases, the purified enzymes and marker proteins were applied to a Superdex^TM^ 200 10/300 GL column (GE Healthcare UK Ltd.). The molecular masses were calculated from the standard curve of marker proteins by extrapolation. The structures of enzymes were speculated from the molecular masses.

### Enzyme activity assays

NHase activity was measured by the increase of product. The reaction mixture (0.5 ml) contained 10 mM KPB (pH 7.4), 200 mM 3-cyanopyridine, and 10 μl of the appropriate amount of the enzyme solution. The reaction was performed at 25°C for 10 min and stopped with the addition of 0.5 ml of acetonitrile. The amount of product formed was determined by measuring the absorbance at 215 nm and extrapolation from a standard curve. One unit (U) of NHase activity is defined as the amount of enzyme that released 1 μmol nicotinamide per minute under these assay conditions.

### Determination and spectrum scanning of cobalt ions in the purified enzymes

The purified enzymes(0.5 mg/ml) were analysed with a Shimadzu AA-7000 atomic absorption spectrophotometer under the following conditions: wavelength, 240.73 nm; lamp current, 12 mA; and slit width, 0.2 nm. The amount of cobalt ion in the purified enzymes was calculated by extrapolation from a standard curve.

UV-V is spectra were obtained with a U-3900 spectrophotometer (Hitachi, Tokyo, Japan) at room temperature. Enzymes were dialyzed against 10 mM KPB (pH 7.5), and 1.0 mg/ml samples were prepared.

### Measurement of the kinetic parameters, thermal stabilities, product tolerance and optimal pH

The kinetic parameters (*K*_m_, *V*_max_, *k*_cat_) of *BAP14K* and the fusion proteins were determined in 10 mM KPB at 25 °C, and the concentration of enzymes was 0.2 mg/ml. The concentrations of 3-cyanopyridine were 10, 20, 50, 100, and 200 mM, and the reaction was terminated with acetonitrile after 2 minutes. Statistical analyses were performed using the Graphpad Prism 5 software package.

To determine the thermal stability, we incubated the purified enzymes at 50°C for 40 minutes and measured their activity every ten minutes. The method of activity determination was as mentioned above.

The product tolerance was represented by the decrement of 3-cyanopyridine in the presence of 0.5 M nicotinamide, and without nicotinamide was used as the control. To compare the product tolerance of the recombinases, we determined the reduction of 3-cyanopyridine in the mixtures with and without nicotinamide.

The optimal pH for enzyme activity was determined by performing the assay in pH buffers (KH_2_PO_4_, 3.893 g/l; C_6_H_8_O_7_·H_2_O, 6.008 g/l; H_2_BO_3_, 1.769 g/l; and Barbital-Na_2_, 5.266) (pH 6.0 to 11.0). The pH with the highest activity was defined as the optimal pH, and the highest activity was taken as 100%.

### CD analysis

Circular dichroism (CD) spectra were collected using a MOS-450/AF-CD-STP-A (Bio-Logic, Grenoble, France) with a 1-cm path-length quartz cuvette at a protein concentration of 0.2 mg/ml in 10 mM KPB. The spectropolarimeter and xenon lamp were warmed up for 30 minutes before experiments. We measured the ellipticity between 190 and 250 nm, and the spectrum of a buffer blank was subtracted. We used an online method K2D3 on a publicly accessible web server at http://k2d3.ogic.ca/ to predict the secondary structure types. This method improves the predictions, particularly for the wavelength interval between 200 and 240 nm and for beta-strand content^42^.

### Activation of apo-NHase by cobalt-containing α(StrepP14K)_2_

In previous study, the activator P14K could exist with the α-subunit in the form of heterotrimer α(P14K)_2_, and it could exist stably when a Strep-tag was added to the N-terminal of P14K^12^. As a result, we used pET24a-StrepP14K to prepare α(StrepP14K)_2_. The apo-protein was cultured in the absence of cobalt. Conversely, the cobalt-containing protein was cultured in the presence of cobalt. The purified apo-NHase (0.1 mg/ml) was mixed with the purified cobalt-containing α(StrepP14K)_2_ (0.8 mg/ml) followed by incubation in 10 mM KPB (containing 0.5 mM dithiothreitol, pH 7.4) at 25°C. As a control, the purified apo-NHase was mixed with cobalt ion (20 μM) in the same manner.

### Mass spectrometry analysis

MALDI-TOF mass spectrometer (ultrafleXtreme, Bruker Daltonics) was used to determine the molecular mass of the fused subunit. The recently introduced MALDI-TOF mass spectrometers allow the acquisition of high-quality fragment ion spectra from biomacromolecule by laser-induced fragmentation (LID). Matrix (7.6 mg 2,5-Dihydroxy acetophenone was diluted in 375 μl ethanol and add 125 μl of 18 mg/ml ammonium dihydrogen citrate solution) was first prepared, and 2 μl Matrix, 2 μl 2% TFA solution and 2 μl sample (5 mg/ml) were mixed. The sample solution was mixed fully using pipette tips until the crystallization could be observed. The 0.5-μl-sample solution was spotted and dried on a polished stainless steel target plate. Quantification of mass signals was performed by FlexAnalysis software.

### Identification of the modification of Cys

The sample treatment is following a commonly used protocol^43^. In brief, the sample solution was first denatured in 8 M urea, with disulphide bonds reduced by dithiothreitol and all Cysteine residues were carboxamidomethylated by iodoacetamide. The sample was then cleaned by dialysis, and digested with TPCK-trypsin (Promega) in the digestion buffer (100 mM ammonium bicarbonate, pH8.5). The peptides from the digestion were completely dried down in a SpeedVac device (Thermo). The dried sample was then re-dissolved in sample solution (2% acetonitrile, 0.5% formic acid and 97.5% water). A dissolved peptide sample was then analysed by a NanoLC-ESI-MS/MS system.

NanoLC-ESI-MS/MS analysis of digested protein samples was carried out by a high performance liquid chromatography (HPLC) system (Agilent) with a reverse phase C18 column of a 75 micrometre inner diameter and 8 cm in length. The injection time was 20 minutes. The HPLC Solvent A was 97.5% water, 2% acetonitrile and 0.5% formic acid, and Solvent B was 9.5% water, 90% acetonitrile and 0.5% formic acid. The gradation time was 100 minutes from 2% Solvent B to 90% solvent B, plus 20 minutes for sample loading and 20 minutes for column washing. The column flow rate was approximate 800 nanoliter per minute after splitting. Typical injection volume was 3 μl. The HPLC system was on-line coupled with LTQ mass spectrometer (Thermo) in a way of a sample eluted from HPLC column was directly ionized by an electrospray ionization (ESI) process and enter into the mass spectrometer. The ionization voltage was optimized each time and normally in a range of 1.2 kV–1.8 kV.

## Additional Information

**How to cite this article**: Xia, Y. *et al.* Construction of a subunit-fusion nitrile hydratase and discovery of an innovative metal ion transfer pattern. *Sci. Rep.* **6**, 19183; doi: 10.1038/srep19183 (2016).

## Supplementary Material

Supplementary Information

## Figures and Tables

**Table 1:** Characterization of the purified recombinant NHase variants.

	Specific activity (U/mg)	*K*_m_^a^ (mM)	*k*_cat_ (s^−1^)	*k*_cat_/*K*_m_ (∙10^3^s^−1^M^−1^)	Cobalt content (mol of ions/mol of protein)
Wild-type NHase	324.8 ± 9.6	41.2 ± 7.7	335.1 ± 57.6	8.1	0.78 ± 0.01/αβ
NHase-(BA)	69.1 ± 3.2	65.8 ± 10.6	135.8 ± 8.9	2.1	0.12 ± 0.01/(βα)
NHase-(BA)P14K	499.2 ± 14.0	61.3 ± 7.3	723.4 ± 32.8	11.8	0.84 ± 0.03/(βα)
NHase-(BAP14K)	452.5 ± 8.2	121.8 ± 21.8	676.5 ± 61.3	5.6	0.82 ± 0.02/(βαP14K)
apo-NHase	2.6 ± 0.3	N.T.	N.T.	N.T.	0.02 ± 0.01/αβ
apo-NHase-(BA)	1.5 ± 0.2	N.T.	N.T.	N.T.	0.03 ± 0.01/(βα)
apo-NHase-(BA)P14K	4.3 ± 0.8	N.T.	N.T.	N.T.	0.03 ± 0.01/(βα)
apo-NHase-(BAP14K)	4.3 ± 0.5	N.T.	N.T.	N.T.	0.02 ± 0.01/(βαP14K)
R-wild-type NHase	437.6 ± 18.3	N.T.	N.T.	N.T.	0.85 ± 0.04/αβ
R-NHase-(BA)	147.2 ± 3.7	N.T.	N.T.	N.T.	0.23 ± 0.01/(βα)
R-NHase-(BA)P14K	240.5 ± 10.6	N.T.	N.T.	N.T.	0.61 ± 0.02/(βα)
R-NHase-(BAP14K)	219.2 ± 3.9	N.T.	N.T.	N.T.	0.62 ± 0.04/(βαP14K)

The values represent the means ± S.D. for three independent experiments. N.T., not tested.

^a^the kinetic parameters were performed in 10, 20, 50, 100, 200 mM 3-cyanopyridine at 25 °C for 2 min.
