# Supplementary material for: Construction of a subunit-fusion nitrile hydratase and discovery of an innovative metal ion transfer pattern
Source: Sci Rep. 2016 Jan 12;6:19183. doi: 10.1038/srep19183 (PMC4709657; doi:10.1038/srep19183)
Supplement: Supplementary Information [file srep19183-s1.doc]

**Supplementary Information for**

**Construction of a subunit**-**fusion nitrile hydratase and discovery of an innovative metal ion transfer pattern**

Yuanyuan Xia^a^, Wenjing Cui^a^, Zhongmei Liu^a^, Li Zhou^a^, Youtian Cui^a^, Michihiko Kobayashi^b^ *, Zhemin Zhou^a^ *

**Table S1 Oligonucleotide primers used in this study.**

| Primers | Sequence (5’-3’) | Restriction sites |
| --- | --- | --- |
| B-*Nde* I-up | GGAATTC***CATATG***AATGGCATTCACGATAC | *Nde* I |
| P-*Hin*d III-down | GCCC***AAGCTT***TCAAGCCATTGCGGCAACGA | *Hin*d III |
| A-*Hin*d III-down | GCCC***AAGCTT***TCAATGAGATGGGGTGGGTT | *Hin*d III |
| Linker1-up | TACCTGGAGCCAGCGCCAGGTGGGCAATCACACACGCAT |  |
| Linker1-down | CGTGTGTGATTGCCCACCTGGCGCTGGCTCCAGGTAGTC |  |
| Linker2-up | CCCACCCCATCTCATCCAAATGGAGATATAGATATG |  |
| Linker2-down | CATATCTATATCTCCATTTGGATGAGATGGGGTGGG |  |
| B(P)A-up | GTGGGATGACTACCTGGAGCCAGCGATGAAAGACGAACGG |  |
| B(P)A-down | GGTGGTCATGCGTGTGTGATTGCCCAGCCATTGCGGCAAC |  |
| (P)BA-up | CGGCCTGGTGCCGCGCGGCAGCCATATGAAAGACGAACGG |  |
| (P)BA-down | CGCCAGTATCGTGAATGCCATTCATAGCCATTGCGGCAAC |  |

Restriction sites are in italics/bold; the overlapping nucleotides are in underlined.


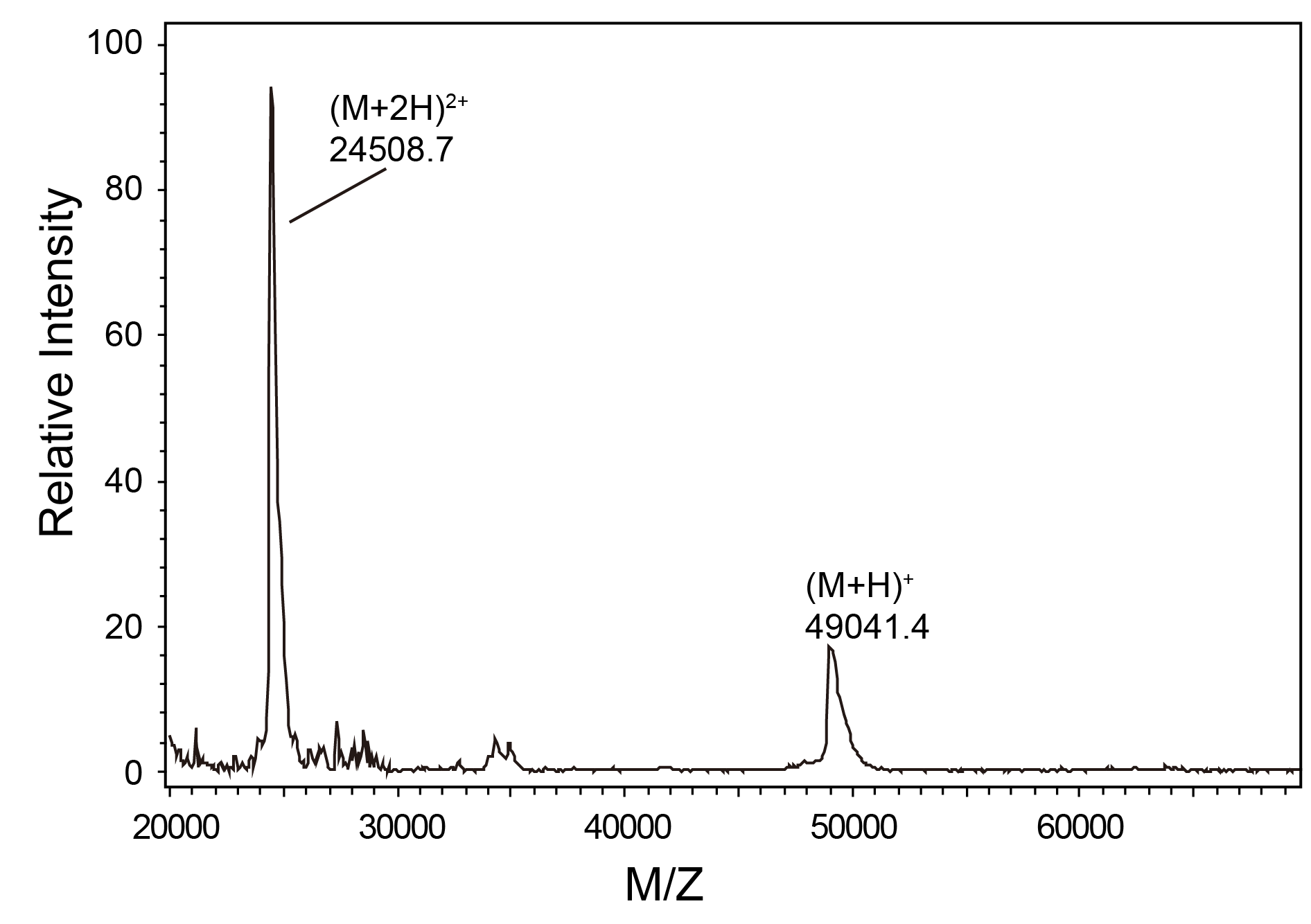


**Fig. S1. MALDI-TOF mass spectra of the NHase-(BA)P14K.**

The mass peaks with the *m/z* 24508.7 and the *m/z* 49041.4 were observed, which correspond to the [M+2H]^2+^ ion and [M+H]^+^ ion of βα (the calculated βα mass: 49241.7), suggesting that NHase-(BA)P14K is the full length of βα.


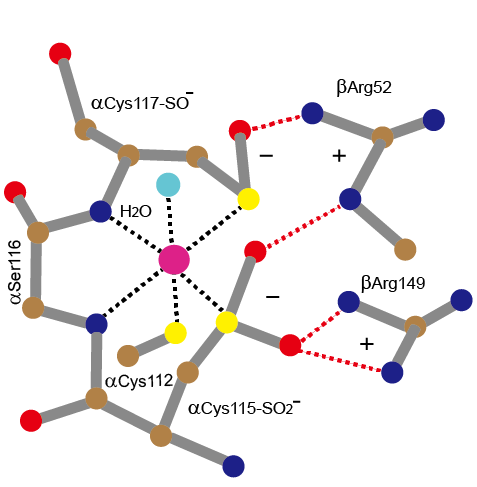


**Fig. S2. A model of the non-corrin cobalt centre of NHase.** The model is based on all known crystal structures of Co-type NHases (1, 2) and Fe-type NHases (3, 4). Atoms are shown in different colors: pink for Co, brown for C, red for O, yellow for S, and blue for N. The salt-bridge networks formed between the modified cysteine and the two-arginine residues are shown as red dotted lines.


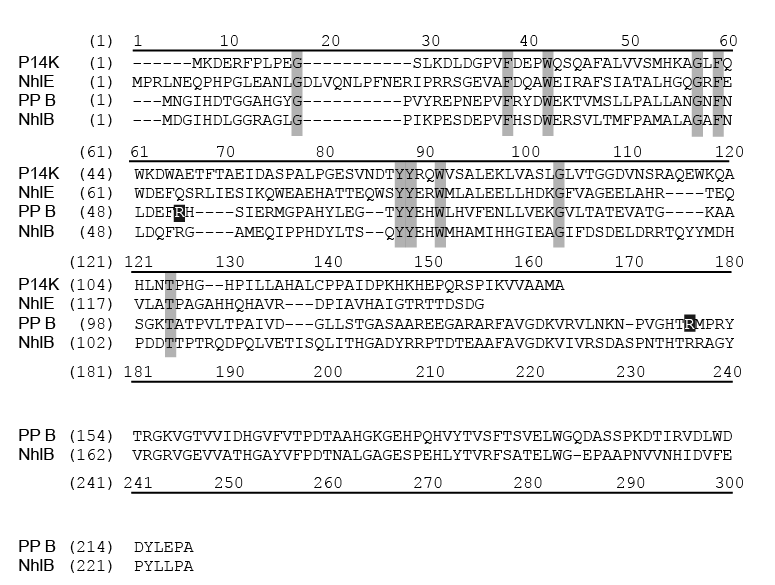


**Fig. S3. Amino acid sequence alignment of P14K, NhlE, and β-subunit of NHases from *P. putida* NRRL-18668 and *R. rhodochrous* J1.**

P14K, activator for NHase from *P. putida* NRRL-18668; NhlE, self-subunit swapping chaperone for L-NHase in *R. rhodochrous* J1; PPB, β-subunits of NHases from *P. putida* NRRL-18668; NhlB, β-subunits of NHases from *R. rhodochrous* J1. The amino acid residues conserved in the four proteins are shown in gray background, the two arginine of β-subunit in active center are shown in black background.
